# *Salmonella* in Broiler Litter and Properties of Soil at Farm Location

**DOI:** 10.1371/journal.pone.0006403

**Published:** 2009-07-28

**Authors:** Victoriya V. Volkova, R. Hartford Bailey, Robert W. Wills

**Affiliations:** 1 Epidemiology Group, Centre for Infectious Disease, School of Biological Sciences, University of Edinburgh, Ashworth Laboratories, Edinburgh, United Kingdom; 2 Department of Pathobiology and Population Medicine, College of Veterinary Medicine, Mississippi State University, Mississippi State, Mississippi, United States of America; University of Oxford, United Kingdom

## Abstract

Contamination of litter in a broiler grow-out house with *Salmonella* prior to placement of a new flock has been shown to be a precursor of the flock's *Salmonella* contamination further down the production continuum. In the southern USA, broiler grow-out houses are primarily built on dirt pad foundations that are placed directly on top of the native soil surface. Broiler litter is placed directly on the dirt pad. Multiple grow-out flocks are reared on a single litter batch, and the litter is kept in the houses during downtime between flocks. The effects of environmental determinants on conditions in broiler litter, hence *Salmonella* ecology within it, has received limited attention. In a field study that included broiler farms in the states of Alabama, Mississippi and Texas we assessed *Salmonella* in broiler litter at the end of downtime between flocks, i.e. at the time of placement of a new flock for rearing. Here we utilized these results and the U.S. General Soil Map (STATSGO) data to test if properties of soil at farm location impacted the probability of *Salmonella* detection in the litter. The significance of soil properties as risk factors was tested in multilevel regression models after accounting for possible confounding differences among the farms, the participating broiler complexes and companies, and the farms' geographical positioning. Significant associations were observed between infiltration and drainage capabilities of soil at farm location and probability of *Salmonella* detection in the litter.

## Introduction

Newly hatched broilers are highly susceptible to *Salmonella* colonization, likely due to the composition of their intestinal microbiota [1]–[4]. Over the last 50 years in the southern USA, grow-out broilers in intensive production systems have been housed on deep litter on the floor. In these economy-of-scale production systems, the birds are placed into grow-out houses within a day after hatch, directly on litter. Therefore, if *Salmonella* is present in the litter, the birds are exposed at a time when they are highly susceptible. In fact, the presence of *Salmonella* in the grow-out house, specifically in the litter, prior to placement of a new flock and contamination of the previous flock reared in the house have been shown to be precursors of higher *Salmonella* frequencies in the new flock at later stages of the production continuum [5]–[7]. We have also observed that higher *Salmonella* contamination of the litter at the time of flock placement was associated with increased probability of *Salmonella* detection on broiler carcasses from the flock at the post-chill point in processing [8]. The role of litter in *Salmonella* cycling in broiler grow-out houses and flocks, and the effects of chemical processes in aging litter and of litter management on *Salmonella* ecology in this matrix have been studied extensively [5], [6], [9]–[23]. However, the question of whether environmental determinants at the location of broiler farm impact on the litter conditions and hence the degree of its *Salmonella* contamination has received limited attention [16], [24]. In the present study, we utilized the U.S. General Soil Map (STATSGO) [25] data to investigate the associations between soil properties at farm location and probability of *Salmonella* detection in the litter at the time of a new flock placement.

## Results

### Description of sampled farms and litter management

All sampled farms were conventional grow-out farms and reared broilers as “all-in/all-out”. The number of broiler houses on a farm ranged from 2 to 16, averaging 5. The litter used was pine shavings. A total of 76 houses were sampled (two on each of 38 farms) within 1 to 2 hours prior to placement of new flocks. Four litter samples (LS) and four drag swabs of the litter (DS) were collected from each house. The length of time the houses were empty after the harvest of previous flocks (i.e. downtime prior to sampling) ranged from 5 to 26 days, averaging 12 days (based on that known for n = 64 houses sampled). During this downtime, in 73 out of the 76 houses the litter was mechanically conditioned by removal of the caked portions, after which fresh pine shavings were added in 20 of the houses. The litter was new in three houses: one sampled farm was new and the two houses were used to grow broilers for the first time, and the litter was totally replaced in the other house. The average age of the current litter in the houses at the time of sampling was 15 months (n = 66); it had been used to grow on average five or six broiler flocks (n = 66). The average age of the previous litter used in the houses was 26 months by the time it was totally cleaned out and replaced with the current litter (n = 52). During the downtime prior to sampling, the current litter received chemical treatment for darkling beetle control by topical application (most commonly 4 days before sampling) in at least 63% of the houses (a single commercially available product was reported in all cases). The litter was treated for ammonia control (usually 1 week before sampling) in at least 25% of cases by topical application of treatment powder (two commercially available products and one household chemical were reported to be used). Both the treatments were applied to the litter in at least 20% of the houses. All these litter management procedures were within the scope of routine practices for the industry. Therefore, acknowledging that any of these procedures could have an impact on *Salmonella* in the litter, we preserved data from all sampled houses in the analyses.

### *Salmonella* in litter

Of the 76 houses sampled, 29% yielded at least one *Salmonella*-positive litter sample. *Salmonella* was detected in all four litter samples collected in 4% of the houses, in three out of four in 6.5%, in two out of four in 2.5%, and in only one litter sample in 16% of the houses. Of the 38 studied farms, 21% had at least one *Salmonella*-positive litter sample from both the houses, 16% of the farms had *Salmonella* present in litter samples from one but not from the other house, and no *Salmonella* was detected in litter samples from either of the two houses on the remaining farms (63%).

For drag swabs of litter, of the 76 sampled houses 38% yielded at least one *Salmonella*-positive swab. All four swabs bore *Salmonella* in 12% of the houses, three swabs out of four in 6.5%, two swabs out of four in 6.5%, and only one swab from 13% of the houses. *Salmonella* was detected in at least one drag swab from both the houses on 26% of the farms, from only one house on 24% of the farms, and was not detected in drag swabs from either house on the remainder (50%).

Examining the agreement between the two sampling techniques, *Salmonella* was detected in at least one litter sample and drag swab in 18% of the houses sampled. *Salmonella* was absent in samples of both types from 51% of the houses. The two techniques disagreed for the rest of the houses (30%) in whether they yielded at least one *Salmonella*-positive sample. Spearman correlation coefficient between the numbers of *Salmonella*-positive litter samples and drag swabs from a house was 0.47 (*p*<0.001) suggesting a moderately strong and statistically significant positive correlation (Volkova V. V., Dazo-Galarneau K., Bailey R. H., Byrd J. A., Wills R. W. Comparison of broiler litter sample and drag swabs to assess *Salmonella* contamination of broiler grow-out houses prior to placement of new flocks. Proceedings of the 87th Annual Meeting of the Conference of Research Workers in Animal Diseases, 3–5 December 2006, Chicago, IL, USA). From 14 farms with at least one *Salmonella*-positive litter sample (from either of the two houses), four yielded no positive drag swabs. From 19 farms where *Salmonella* was detected in at least one drag swab, nine farms had no positive litter samples. We therefore considered that the two sampling techniques provided somewhat different measurements of *Salmonella* contamination of broiler litter in sampled houses. Analyses of associations between the soil properties at farm location and probabilities of *Salmonella* detection in the litter samples and drag swabs were conducted in parallel.

### Location of sampled farms and structure of soil data

Geographical coordinates of sampled farms were recorded during the farm visits (a Global Positioning System unit was normally placed equidistantly between the two houses sampled on a farm). The farms were located between 31–34° north latitude and 87.5–96.5° west longitude in the states of Alabama, Mississippi and Texas. The U.S. General Soil Map (STATSGO) data for these states were downloaded from the website of the Natural Resource Conservation Service, United States Department of Agriculture (NRCS, USDA). The data described the soil properties, land usage, vegetation and wildlife habitat suitability. Information records in the data were structured as follows: i) a soil map unit was the smallest geographical unit and therefore was the unit of analysis, ii) a soil map unit consisted of map unit components: specified kinds of soil or miscellaneous areas (areas with little or no recognizable soil), and iii) a component was formed by layers. Sampled farms were located within 25 soil map units. A soil property was described either for a layer or for a component; the data were aggregated to the soil map unit level following guidance in the STATSGO Data User Information Manual. A total of 53 variables were considered at the screening step of analyses.

### Results of analyses for individual soil properties

A number of the soil properties at farm location were associated in the screening step of analyses (*p-*value≤0.150 as a single fixed effects factor in the basic model, design of the basic model is outlined in *Materials and Methods*) with probabilities of detecting *Salmonella* in the litter samples or drag swabs or both (Table 1). Starting with those associated with both outcomes, higher total water capacity of the soil profile (the total water that can be stored) appeared to be a protective determinant. Similarly, protective effects were observed for soils with higher pH. Soils with higher tolerance to erosion in terms of T-factor (the maximum rate of soil erosion permitting a high crop productivity) also appeared to be protective. In contrast, soils with a higher percentage by weight of rock fragments greater than 7.6–25.4 cm in diameter were associated with increased probabilities of *Salmonella* detection in the litter samples and drag swabs. An increase in the percentage of soils rated as hydric in the map unit was also associated with higher probabilities to detect *Salmonella* in samples of both types.

**Table 1:** Soil properties at broiler grow-out farm location associated with probabilities of detecting *Salmonella* in the samples and drags swabs of litter in screening analyses (n = 76)^a^.

				Litter samples		Drag swabs of litter	
Risk factor	Response	Mean (range) or count of sampled houses	Increment modelled	OR (95% CI) for increment or to reference category	*p-*value	OR (95% CI) for increment or to reference category	*p-*value
Total available water capacity	cm	25.85 (19.88–32.05)	1.00 cm	0.59 (0.44, 0.79)	0.0007	0.74 (0.57, 0.95)	0.0192
Soil pH	pH	4.99 (4.69–6.09)	0.25	0.06 (0.01, 0.29)	0.0010	0.40 (0.14, 1.17)	0.0915
Moist bulk density	g/cm^3^	1.48 (1.34–1.58)	0.10 g/cm^3^	2.94 (0.66, 13.10)	0.1524^b^	-	-
Erodibility K-factor	K-factor, numerical	0.29 (0.21–0.37)		0.79 (0.64, 0.98)	0.0356	-	-
Tolerance to erosion T-factor	Tonne	4.00 (3.00–5.00)	1.00 tonne	0.21 (0.04, 1.10)	0.0643	0.24 (0.04, 1.36)	0.1042
Wind erodibility group (WEG)	WEG-group, numerical	4.00 (2.00–6.00)		0.45 (0.16, 1.27)	0.1299		
Rock fragments greater than 7.6–25.4 cm in diameter	% by weight of soil	0.37 (0.00–1.95)	0.25%	1.05 (1.00, 1.11)	0.0662	1.07 (1.02, 1.13)	0.013
Material<7.6 cm in diameter	% by weight of soil	97.67 (87.04–100.00)		0.78 (0.57, 1.06)	0.1056	-	-
Material<2 mm in diameter (range in clay content)	% by weight of soil	19.70 (14.42–29.82)		-	-	0.82 (0.62, 1.07)	0.1408
Percentage of soils of hydrologic group B (silt loam, loam)	%	35.25 (0.00–56.00)	10%	-	-	0.63 (0.39, 1.00)	0.0503
Ratio of percentages of soils of hydrologic groups A&B (lower run-off potential) to those of groups C&D (higher run-off potential)	Ratio	0.75 (0.00–2.00)		-	-	0.16 (0.03, 1.03)	0.0539
Natural drainage rate class^c^	Ordered class	3 (1–5)	1 class	-	-	0.22 (0.07, 0.69)	0.0107
Percentage of soils rated as hydric	%	14.76 (0.00–71.00)	10%	1.48 (0.96, 2.28)	0.0715	1.95 (1.26, 3.02)	0.0037
Latest month annual flooding can start in a normal year	Jan Nov/Dec	60 16		6.41 (0.53, 77.90) Reference	0.1401	5.46 (0.57, 52.30) Reference	0.1362
Earliest month annual flooding can end in a normal year	Apr/May Before or in March	30 46		0.27 (0.05, 1.49) Reference	0.1293	-	-
Latest month annual flooding can end in a normal year	May/June Before or in April	50 26		10.05 (1.32, 83.80) Reference	0.0271	3.85 (0.60, 24.80) Reference	0.1516^b^
Proportion of soils with perched water table^d^	Ordered class	3 (1–5)	1 class	-	-	0.53 (0.26, 1.09)	0.0814
Primary farm land classification (as defined in STATSGO)	Ordered class	2 (1–5)	1 class	0.03 (0.002, 0.46)	0.0126	-	-
Suitability to produce the habitat requirements for wetland wildlife^e^	Ordered rating	2 (1–4)	1 order	-	-	3.34 (1.13, 9.84)	0.0301
Suitability to produce the habitat element wetland plants^e^	Ordered rating	2 (1–4)	1 order	-	-	3.63 (1.15, 11.50)	0.0294
Suitability to produce the habitat element shallow water^e^	Ordered rating	2 (1–4)	1 order	-	-	3.31 (1.12, 9.76)	0.0308
Suitability to produce the habitat element shrubs^f^	Good Fair or less	68 8		-	-	0.06 (0.01, 0.52) Reference	0.0126
Suitability to produce the wildlife habitat element coniferous hardwood trees^f^	Good Fair or less	68 8		0.14 (0.01, 1.36) Reference	0.0884	0.16 (0.01, 2.11) Reference	0.1598^b^
Suitability to produce the habitat requirements for woodland wildlife^f^	Good Fair or less	68 8		0.14 (0.01, 1.36) Reference	0.0884	0.16 (0.01, 2.11) Reference	0.1598^b^

a The associations established after accounting for random variability among the broiler farms, complexes and companies, and farm latitude.

b Variable with a marginal significance (*p*≤0.150) in the screening step retained for further analysis.

c Soil natural drainage rate classes: Somewhat Excessive (15.2–50.8 cm per hour)–5; Well (5.1–15.2 cm per hour)–4, Moderately Well (1.5–5.1 cm per hour)–3, Somewhat Poor (0.5–1.5 cm per hour)–2, and Poor (0.2–0.5 cm per hour)–1.

d Proportion of soil map unit components with perched water table classes: 75–100% of components with perched and the rest with apparent water table–class 5, 50–74% of components with perched and the rest with apparent water table–class 4, equal percentages (50%/50%) of components with perched and apparent water tables–class 3, 50–74% of components with apparent and the rest with perched water table–class 2, 75–100% of components with apparent and the rest with perched water table–class 1.

e Soils suitability to produce the habitat for particular vegetation or wildlife rating: good-4, fair-3, poor-2, and very poor-1.

f See *Results* *of analyses for individual soil properties* for how the ratings of soils' suitability to produce the habitat for particular vegetation or wildlife were converted into dichotomous variables.

Of the soil properties associated with probability of detecting *Salmonella* in the litter samples alone, a higher percentage by weight of soil of material less than 7.6 cm in diameter was protective. However, a higher probability of *Salmonella* detection in the litter samples was observed for the soils with a higher moist bulk density (the weight per unit of volume of the soil of material that is less than 2 mm in diameter). Erodibility K-factor is a soil erosion designation that relates to susceptibility of soil particles to detachment and movement by water (K-factor increases with increasing susceptibility to erosion). The soils with a higher K-factor were associated with a lower probability of detecting *Salmonella* in the litter samples. The wind erodibility grouping (WEG) refers to a soil's susceptibility to being blown by wind. Soils less susceptible to wind erosion in terms of the WEG (a higher group order) were associated with reduced probability of *Salmonella* detection in the litter samples.

Of the soil properties associated with probability of detecting *Salmonella* in the drag swabs of litter alone, a higher percentage of soils of hydrologic group B (silt loam or loam, moderate infiltration, and moderate well-to-well drainage) in the soil map unit of farm location was linked to a lower probability of detection. Similarly, better natural drainage of the soils in terms of the natural drainage class (ordered from poor to somewhat excessively drained) was associated with reduced probability of detecting *Salmonella* in the drag swabs. An overall increased ratio of the soils with comparably lower run-off potential (hydrologic groups A and B) to those with comparably higher run-off potential (hydrologic groups C and D) in the map unit was also associated with reduced probability of *Salmonella* detection in the drag swabs. Increased clay content (the percentage by weight of the soil of material less than 2 mm in size) over the components of the map unit was also associated with a lower probability of detecting *Salmonella* in the drag swabs.

The apparent water table is the top zone of saturation in the soil profile. The perched water table is the zone of saturation occurring above the normal water table in a soil in which there is an impermeable layer that separates the perched water table from the permanent ground water. A higher percentage of the map unit components with the perched water table within the soil map unit of farm location was associated with a lower probability of detecting *Salmonella* in the drag swabs. (All of the soil map units studied had only apparent or perched water tables; artesian water table was not encountered).

In terms of the surface soil texture, no associations were observed between the percentage of clay, silt or sand in the surface soil texture of the map unit where the farm was located and probability to detect *Salmonella* in either litter samples or drag swabs (*p-*value>0.150 for each of the percentages as a single fixed effects factor in the basic model for either outcome).

Interestingly, the time of year when natural annual flooding could occur in a normal year within the soil map unit where the farm was located was associated with probabilities of detecting *Salmonella* in the litter. Higher probabilities of detecting *Salmonella* in both litter samples and drag swabs were observed if annual flooding could occur later in a normal year. In particular, there were higher probabilities of the detection when the latest the flooding could start was January rather than in November or December of the preceding year; also, if the latest the flooding could end was May or June rather than before or during April. However, the relationship between the timing of annual flooding and probabilities of *Salmonella* detection appeared to be more complex; when the earliest that annual flooding could end was April or May rather than before or during March, this was associated with reduced probabilities of *Salmonella* detection in both litter samples and drags swabs. Investigation of the timing of annual flooding as a risk factor did not imply that the sampled houses were built within flood-prone areas in local topology, as the sample collection was carried out at different times of year the former was not evaluated. However, during sampling visits we recorded (for n = 74 houses) if the sampled house was placed on a hill (at a visually defined relatively high point in local topology), under a hill (at a visually defined relatively low point in local topology) or neither (no visually observable difference between the house location and elevation of the surrounding areas). Fourteen sampled houses were placed on a hill, eight under a hill, and neither could be defined for the remaining 52 houses (n = 74 recorded). No statistically significant associations were detected between this factor and probabilities of detecting *Salmonella* in the litter samples or drag swabs.

While farmland classifications of soils differ, they generally express the qualities of soils at the location in terms of soil temperature, moisture, soil pH, water movement, growing season and other factors. A higher category of the “primary farm land classification” given in the STATSGO for the soil map unit of farm location was associated with reduced probability of detecting *Salmonella* in the litter samples.

A higher probability of *Salmonella* detection in the drag swabs was observed with the soils in the map unit of farm location being better suited to produce shallow water habitat element, habitat element for wetland plants or habitat for wetland wildlife. In contrast, suitability of the soils to produce better habitat for coniferous hardwood trees and woodland wildlife (the two ratings were completely collinear) was associated with lower probabilities to detect *Salmonella* in both litter samples and drag swabs. Good suitability of the soils to produce the habitat element shrubs was associated with a lower probability of *Salmonella* detection in the drag swabs.

### Final models of soil properties at broiler farm location associated with probabilities of detecting *Salmonella* in the samples and drag swabs of litter

The final model demonstrated that three soil properties at farm location were most strongly associated with probability of detecting *Salmonella* in the samples of litter (Table 2). In particular, a higher probability of *Salmonella* detection was observed if annual flooding at the location in a normal year could start later (January rather than the previous November or December), and with a higher moist bulk density of the soils. While a higher total water capacity of the soils was a protective determinant.

**Table 2:** Fixed effects risk factors in the final models of soil properties at broiler grow-out farm location associated with probabilities of detecting *Salmonella* in the samples and drags swabs of litter (n = 76)^a^.

Outcome/risk factor	Response	Mean (range) or count of sampled houses	Increment modelled	OR (95% CI) for increment or to reference category	*p-*value
Litter samples outcome					
Total available water capacity	cm	25.85 (19.88–32.05)	1 cm	0.56 (0.43, 0.75)	0.0002
Moist bulk density	g/cm^3^	1.48 (1.34–1.58)	0.10 g/cm^3^	3.83 (1.03, 14.22)	0.0454
Latest month annual flooding can start in a normal year	Jan	60		9.42 (1.03, 86.33)	0.0473
	Nov/Dec	16		Reference	
Drag swabs of litter outcome					
Tolerance to erosion T-factor	Tonne	4.00 (3.00–5.00)	1.00 tonne	0.05 (0.01, 0.28)	0.0012
Rock fragments greater than 7.6–25.4 cm in diameter	% by weight of soil	0.37 (0.00–1.95)	0.25%	1.06 (1.01, 1.11)	0.0256
Natural drainage rate class^b^	Ordered class	3 (1–5)	1 class	0.13 (0.04, 0.42)	0.0012

a Random effects of the broiler farms, complexes, companies, or farm latitude were not found to make significant (*p*≤0.050) contributions to the variability in the responses in these models.

b Soil natural drainage rate classes: Somewhat Excessive (15.2–50.8 cm per hour)–5, Well (5.1–15.2 cm per hour)–4, Moderately Well (1.5–5.1 cm per hour)–3, Somewhat Poor (0.5–1.5 cm per hour)–2, and Poor (0.2–0.5 cm per hour)–1.

The final model developed for the drag swabs of litter (Table 2) showed that probability of detecting *Salmonella* in this sample was most strongly affected by: i) the increased risk due to a higher percentage by weight of rock fragments (greater than 7.6–25.4 cm in diameter) in the soils; and by the protective effects of ii) increased tolerance to erosion (as expressed by T-factor), and iii) improved natural drainage capabilities of the soils.

These associations were established after accounting for potential confounding differences among: the sampled farms, production complexes or companies managing broiler flocks on the farms, and farm latitude.

## Discussion

Although the present analysis was exploratory in its nature, the results suggest that certain properties of soils at broiler farm location may be impacting the conditions of litter in the houses, which are reflected in the degree of litter contamination with *Salmonella*. Soil consists of mineral matter, organic matter and pore space; porosity of the soil and water movement through it depend on the soil structure, texture of mineral matter, amount of organic matter, and the patterns of soil compaction and disturbance. Those soil properties associated with probability of detecting *Salmonella* in the broiler litter in this study were primarily the soil texture (relative proportions of particles of different sizes) and the infiltration and drainage capabilities, i.e. the properties defining the pattern of water movement through the soil profile. The results suggest that a lower degree of broiler litter contamination with *Salmonella* may be related to the farms being built on soils with better natural drainage but also with higher available water capacity (amount of water that can be stored). Connectively, these are the soils with a lower content of large (>7.6 cm in diameter) rock fragments, which reduce the available water unless the rocks are porous, and a lower moist bulk density (the weight per unit of volume of the material <2 mm in diameter), which controls the pore space otherwise available for water. The effects of soil properties on the litter conditions are further complicated by interactions with local climate. In particular, a later season of natural annual flooding in a normal year is associated with a higher probability of detecting *Salmonella* in the litter in broiler houses built on dirt pad foundations across the year (broiler houses in this study were each sampled once during the four-year study period). This hypothesis requires further investigation that incorporates more detailed climatic information.

One possible explanation for the associations observed in this study is that the soils' drainage capacity directly influences the moisture level in the deep litter on the floor of broiler houses. While we did not measure moisture level in the litter samples collected, the moisture level and water activity in broiler litter are known to impact *Salmonella* ecology in this matrix [13], [16], [19], [26].

Alternative or synergistic effects may be due to the role of soil in determining the risks of *Salmonella* introduction into broiler houses on mechanical vehicles or with living reservoirs. For the former, characteristics of the soil at broiler farm location may impact on the risks of *Salmonella* being brought into the houses on such mechanical vehicles as farm-worker footwear or movable equipment. For the latter, it is plausible that properties of the soil determine the species composition and distribution of rodents infesting the farm. A field study undertaken in Argentina demonstrated that rodents show habitat selection on poultry facilities on a farm and a shed within the farm level [27]. To the best of the authors' knowledge, no data is available on whether the habitat selection by rodents is affected by types of the soils on the farm. There is also contrasting evidence as to whether rodents constitute an important source of *Salmonella* on broiler farms. Observation of rodents by the farmer was indicative of *Salmonella* persistence in grow-out broiler houses after decontamination between sequential flocks in a study in France [24]. However, in another observational study in the southern USA, *Salmonella* recovery from mice samples obtained on grow-out broiler farms was comparably low [28].

The associations detected in this study were observed in grow-out broiler houses built on dirt pad foundations placed directly on top of the native soil surface. First, these relationships may not hold in broiler houses constructed differently. Second, the pad is made of intensively compacted dirt, often with a high percentage of clay, and is likely to have different drainage characteristics than the native soils under and around the broiler house. Any effect of the soil properties on the litter conditions is therefore mediated by the pad. The extent of this impact likely depends on original qualities of the dirt pad, the soil properties, and how the dirt pad deteriorates over time under forces of the latter and climatic conditions.

In this study the broiler litter was sampled at the end of a downtime in-between the flocks sequentially reared in grow-out houses (except one new farm). The only litter used was pine shavings. Different materials are used as broiler litter in intensive broiler production systems around the world [4], [7], [9], [24], [29], [30]. Often it is plant-origin by-products of other industries (forestry or food crop production), as in the case of pine shavings. The relationships between *Salmonella* in the litter and properties of soil at the farm location observed here may not hold for other litter compositions.

The results of present study may be susceptible to ecological fallacy. Aggregated data on properties of soils within the soil map unit where the farm was located were analysed rather than the precise characteristics of the soils immediately underlying or surrounding the broiler houses; it was unknown how well the former exemplified the latter.

In conclusion, properties of soil at the broiler farm location may impact the conditions of floor litter in the houses; in turn, the litter conditions determine the ecology of *Salmonella* and potentially that of other bacteria of food safety or poultry health importance in this matrix. The long-term effects of such associations require an investigation in which the soil properties, other on-farm conditions and climatic determinants are co-examined. Usefulness of the information derived in terms of the soil properties as risk factors will depend on the cost-effectiveness of incorporating considerations of the soil properties into the site selection for a broiler farm versus the subsequent control of *Salmonella* on the farm. Alternative foundations for broiler grow-out houses (rather than the dirt pads placed directly on top of the native soil surface) may be worthy of consideration.

## Materials and Methods

### Sample collection and processing

Sample collection continued from 2003 to 2006 and included 38 conventional broiler grow-out farms in the states of Alabama, Mississippi and Texas. The 38 farms were operating within 10 broiler production complexes belonging to two broiler companies. Random selection of the farms for inclusion in the study was not deemed feasible. The farms were selected by the participating companies (so that the flocks to be placed after collection of the litter samples and drag swabs when grown would be processed on a Monday or Tuesday, to facilitate processing of further samples collected for other research goals). Therefore a selection bias might have been introduced. Compliance of the growers selected was absolute. Despite the convenience sampling, we consider that the houses sampled were generally representative of conventional broiler farms in the southern USA during the years of study.

On each farm, two houses were sampled, usually an end house and the adjacent one, for a total of 76 houses. The lengths of the sampled houses were 110 to 152 m, most commonly 128 m or 152 m. Half of the sampled houses were 12 m wide and the other half were 13.4 m wide. Customary to the region, all sampled houses were built on dirt pad foundations placed directly on top of the native soil surface, and with the long side oriented east to west.

Each house was sampled once: within 1 to 2 hours prior to placement of the new flock. Four litter samples (LS) were obtained per house. Each litter sample consisted of eight individual portions of litter collected equidistantly along one of four lines parallel to the long side of the house and then pooled into a Whirl-Pak® Bag (NASCO, Fort Atkinson, WI). Four drag swabs (DS) were collected by dragging two swabs along two lines parallel to the long side of the house on one side of the house, and then repeating the sampling on the other side of the house. The drag swabs were prepared, collected and processed as previously described [17], [31]–[33]. Briefly, each swab was made with 10.2×10.2 cm cotton gauze (Abco Dealers, Inc., Nashville, TN). A swab was tied to 182.9 cm of cotton-polyester string (The Lehigh Group, Macungie, PA). The swab and string were steam-sterilized and aseptically transferred into a sterile Whirl-Pak® Bag containing 20 mL sterile double strength skimmed milk. The latter was prepared according to the manufacturer's instructions (Wal-Mart Stores, Inc., Bentonville, AR), but with double the concentration of milk powder to water (i.e. 91 g per 500 mL). Samples were transported to the laboratory on wet ice. Upon arrival at the laboratory, within 8 hours of the sample collection, 25 grams of each litter sample were placed into a Whirl-Pak® Filter Bag (NASCO, Fort Atkinson, WI), 225 mL of buffer peptone water (BPW) were added, mixed for 1 minute, and incubated at 42°C overnight. To each drag swab sample, 100 mL of BPW were added, the bag was mixed, and the sample was incubated at 42°C overnight.

### *Salmonella* isolation and identification

*Salmonella* isolation from the samples was performed similarly as described by Rybolt *et al.* [33]. In short, after overnight incubation, one mL from each sample was transferred to nine mL of Tetrathionate (TET) broth (Remel Inc., Lenexa, KS), vortexed and incubated at 42°C for 48 hours. After incubation, 0.1 mL of the TET was transferred to 9.9 mL of Rappaport-Vassiliadis (RV) broth (DIFCO Laboratories, Detroit, MI) and incubated at 42°C overnight. After incubation, one loopful of the RV was plated onto a Xylose-Lysine-Tergitol 4 (XLT4) agar plate (Remel Inc., Lenexa, KS), incubated at 37°C overnight, and the plates were examined for *Salmonella*-like colonies. A single colony was picked from a positive XLT4 plate; *Salmonella* identity was confirmed biochemically on Triple Sugar Iron and Lysine Iron Agar slants. *Salmonella* isolation was further confirmed by a slide agglutination assay using *Salmonella* O Antiserum Poly A-I & Vi (DIFCO Laboratories, Detroit, MI) as described by the manufacturer.

### Soil properties' data processing

As mentioned in *Results*, the U.S. General Soil Map (STATSGO) data for the states in which sampled farms were located were downloaded from the website of the Natural Resource Conservation Service, United States Department of Agriculture (NRCS, USDA). The structure of information records was: a soil map unit (the smallest geographical unit and the unit of analysis) consisting of soil map unit components (specified kinds of soil or miscellaneous areas with little or no recognizable soil), with a component formed by layers. The delineations depict the dominant soils in the landscape. The minimum area delineated is approximately 6.2 km^2^ (1,544 acres). Locations of sampled farms were related to the corresponding STATSGO soil map units using ArcView® (Environmental System Research Institute (ESRI), Redlands, CA, USA). Sampled farms were located within 25 soil map units. The information for these soil map units was extracted from three STATSGO tables: Layer, Component and Wildlife Habitat Suitability Tables. Descriptions of individual variables in the Tables were obtained from the STATSGO Data User Information Manual. The STATSGO Layer and Component Tables contained information on the soil properties measured on a given layer or component level, respectively. The data in the Wildlife Habitat Suitability Table provided the habitat information for the soil map unit components.

For the analysis, the values for soil properties expressed in the Tables as numerical or ordered variables were aggregated to the soil map unit level as summary statistics, calculated following the guidance in the User Manual. In particular, from the Layer Table, the total available water capacity was averaged for each layer, then weighted sums were calculated for all the components, and the weighted sum for the soil map unit was obtained. For the other variables in the Layer Table, averages over the layers for each map unit component were calculated, and the weighted average over the components for the soil map unit was obtained. For the soil properties described in the Component Table as numerical or ordered variables, the average values or orders for the map unit components were taken, from which the weighted average for the soil map unit was calculated.

The soil properties qualitatively described in the Component Table were processed individually. Several aggregate variables were developed for the purpose of the analysis and are described here: i) Soils in each map unit component were defined as hydric or not. In general, a hydric soil has been formed under conditions of saturation, ponding or flooding during the growing season for a sufficiently long time to develop anaerobic conditions in the upper part. This rating was summarized as a percentage of the soils defined as hydric from the total in the soil map unit. ii) The soils were grouped into hydrologic groups, from A to D, in order of decreasing infiltration rate, which corresponded to increasing run-off potential. The percentages of components with soils of each hydrologic group from the total components in the soil map unit were calculated for each investigated soil map unit. iii) Soil drainage classification roughly represents the degree, frequency and duration of the wet period. There are seven classes of natural drainage, but only soils of five of these classes were encountered within the soil map units studied. Drainage class was expressed as an ordered variable, with five levels ranging from ‘somewhat excessive’ to ‘poor’ (Somewhat Excessive–5, Well–4, Moderately Well - 3, Somewhat Poor–2, and Poor - 1). The proper levels were assigned to each soil map unit component, and the weighted average over the components for the soil map unit was calculated. iv) Natural flooding is the condition where flowing water from a combination of sources (such as run-off from surrounding areas with higher slopes or water streams overflowing their banks after rains or snow melts) temporarily covers the soil surface. In STATSGO data the estimates of the time of year when natural flooding occurred in a soil map unit were based on interpretation of the regional soil properties and evidence collected during actual NRCS field surveys. Using these data, the variables of the earliest months and the latest months in which natural annual flooding could begin and end in the map units studied in a normal year were developed.

The USDA surface soil texture classifications for the components in the studied soil map units were extracted from STATSGO; percentages of clay, silt and sand for each soil type encountered were approximated with the soil textural triangle. Weighted averages of the clay, silt and sand percentages in the surface soil texture were calculated for each of the map units investigated.

As mentioned, the records in Wildlife Habitat Suitability Table were for the soil map unit components. Based on their properties, the soils were rated in terms of their suitability to produce habitat elements for different vegetation and habitat requirements for different wildlife. Each rating was converted into an ordered variable, with four levels ranging from ‘good’ to ‘very poor’ (Good-4, Fair-3, Poor-2, and Very Poor-1), and the proper level was assigned to a component. Then, the weighted average was obtained for the soil map unit. Additional re-categorization was done for some of these ratings and is indicated in *Results*.

From the three STATSGO Tables, all soil properties were analyzed for which sufficient data were present for the investigated soil map units. There were 50 such variables, which, together with the derived weighted averages of the clay, silt and sand percentages in the surface soil texture, resulted in a total of 53 risk factors screened. We excluded soil properties for which all the data were zeros (soils with such characteristics did not occur in the map units of interest); of the remainder, an additional 9% of variables from the Layer Table and 20% from the Component Table were not eligible for analysis due to the extent of missing data, probably because the soils with such properties were rare in the map units studied. (Given that STATSGO data are in the public domain, we therefore do not list all the variables screened. Taxonomic classification of soils in the map units studied is available upon request).

### Outline of modelling and statistical procedures

The typical broiler production scenario in the southern USA involves company ownership of the breeder, hatching, and processing operations. A company normally consists of a number of production complexes. Via the complexes, the companies control the nutrition, health care, and other aspects of the broiler grow-out. The broilers are grown on contract with privately owned farms. Logistic regression was used to model the relationships between probabilities of detecting *Salmonella* in the litter samples and drag swabs and the risk factors. To account for possible intra-level commonality of unobserved risk factors at each level of the industry's hierarchy [34] and variability among the participating industry units as it relates to *Salmonella* status of the litter in broiler houses, hierarchically structured random effects factors of the companies, complexes and farms were forced into the multi-level risk factor model. The multi-level generalized linear mixed models incorporating the hierarchically structured random effects, and the fixed effects component were fitted using the GLIMMIX procedure in SAS® 9.1 (SAS Institute Inc., Cary, NC). At the start of the analysis, it was determined that as a single fixed effects factor in such a model the latitude of the farm was associated with probabilities of detecting *Salmonella* in both the litter samples and drag swabs (for each 1° north OR = 2.70 for LS, *p* = 0.057; OR = 2.86 for DS, *p* = 0.078). The longitude of the farm was weakly related to *Salmonella* recovery from the litter samples only (for each 1° west OR = 2.27, *p* = 0.1352; for DS *p* = 0.630). The latitude of the farm was added as the fourth random effect to the multi-level model (further designated as the basic model) to adjust for potential spatially-defined confounding factors when testing the soil properties as the risk factors. The soil properties were then tested for associations with *Salmonella* status of the litter as fixed effects factor(s) in the basic model.

Selection of the fixed effects risk factors was done in general following Hosmer and Lemeshow's [35] model building outline. First, at the screening step of analysis for a given outcome (either LS or DS), the significance of each soil property as a single fixed effects factor in the basic model was evaluated, and those associated with the outcome (*p*≤0.1500) were retained for further analysis. Next, all the risk factors retained from the screening were investigated for pair-wise collinearity for a sampled house using statistical methods appropriate for the types of variables. In particular, the numerical and ordered variables were investigated with Spearman correlation coefficient (*ρ*); a pair was considered collinear if the statistically significant correlation (*p*≤0.050) was >|0.6|. The dichotomous factors were investigated with the simple Kappa agreement coefficient. The Kappa statistic was viewed as a measure of agreement between the presence and absence of the two risk factors for a sampled house beyond that occurring by chance; the two were considered collinear if the simple Kappa coefficient with asymptotic *p-*value≤0.050 was >|0.6|. Each case where collinearity was detected was treated separately; this is discussed in further sections. Interaction among the fixed effects risk factors was investigated in the basic model when deemed probable. Then, the fixed effects risk factors remaining in the analysis for the outcome were offered to the basic model all at once, and after each model fit, the fixed effects factor with the highest *p-*value was removed until a model was developed with every fixed effects factor significant at *p*≤0.050.

A limited number of tools are available to evaluate the performance of generalized linear mixed models with different numbers of predictors. In this study, parsimonization of the final model (developed for each outcome through the two-step variable selection outlined above) was pursued, though always preserving the multi-level random effects part of the model. The full and reduced (in terms of the fixed effects factors) models were compared using: i) Generalized Chi-Square/*df* as an approximate measure of the explained residual variation; ii) the Spearman correlation coefficient between the observed and predicted response proportions for a house (considered as an extension of the philosophy of cross-tabulation of the predicted and observed responses used for dichotomous outcomes modelled with logistic regression); and iii) the simple squared deviations statistic (calculated as the sum of [(observed-predicted)^2^] as suggested by Schukken *et al.* [36]).

In the final model adopted for each outcome, significance of contribution of each random effects factor to the variability observed in the response, given the effects of the other random factors and the fixed effects component, was evaluated with a Wald-type test. The test statistic was calculated as [(parameter estimate/parameter standard error)^2^] and assumed to follow a Chi-square distribution with 1 *df* under the null hypothesis.

### Development of final model of soil properties at broiler farm location associated with probability of detecting *Salmonella* in the samples of litter

Of the numerical and ordered risk factors retained for the litter samples outcome from the screening step of analysis, the total water capacity of the soil profile, soil pH, erodibility K-factor and wind erodibility ordered grouping (WEG) were each pair-wise collinear with the primary farmland classification (as defined in the STATSGO) for the soil map unit. Within the former group itself, the K-factor variable was correlated with the total water capacity and the WEG grouping. The farmland classification and the K-factor variables were excluded from further modelling in order to preserve the variables representing more detailed soil characteristics. The total water capacity of the soil profile and its average pH were moderately correlated (*ρ* = 0.74), but both were kept (despite the collinearity) as potentially important soil properties given the goals of the analysis. Of the dichotomous risk factors retained for the litter samples outcome from the screening step, the soil capabilities to produce habitats suitable for coniferous hardwood trees and woodland wildlife were completely collinear; only the former was used further.

Next, the risk factors remaining for the litter samples outcome modelling, were offered to the basic model as the fixed effects factors all at once for the second step of the variable selection (i.e. after each model fit the fixed effects factor with the highest *p-*value was removed until a model with all the fixed effects risk factors significant at *p*<0.050 was obtained). The full model that was developed included three fixed effects variables (Table 2): i) the latest month when annual flooding could start in a normal year (January versus preceding November or December OR = 9.42), ii) the moist bulk density (for each 0.1 g/cm^3^ increase OR = 3.83), and iii) the total water capacity of the soil profile (for each 1 cm increase OR = 0.56). The Generalized Chi-Square/*df* for the full model was 0.54, the Spearman correlation coefficient between the observed and predicted responses was 0.75 (*p*<0.001), and the simple squared deviations statistic was 0.886. Parsimonization of the full model in terms of the fixed effects risk factors was considered; however, dropping any of the three variables led to loss of significance by one of the other two. From the investigated models, a model with an interaction term between the moist bulk density and the total water capacity of the soil profile (*p* = 0.004) demonstrated high correlation between the observed and predicted responses (*ρ* = 0.77) and small simple squared deviations statistic (0.853). However, the factor of the latest month when annual flooding could start in a normal year was non-significant (*p* = 0.200), but dropping this variable diminished the performance of the model. Therefore the full model developed by the two-stage variable selection (Table 2) was adopted as the final model for the litter samples outcome.

The Pearson-type residuals from the adopted final model were plotted against the observed responses and the predicted responses (the latter computed on the scale of the data and incorporating the random effects). LOESS smoothing regression of the residuals on the predicted and the observed responses was used to further visualise possible trends in the residuals. Then, the predicted responses were plotted against the observations. These diagnostic plots suggested that the predictions from the adopted final model tended to overestimate the responses when the observed outcomes were ≤0.25. This might have been a drawback of the statistical procedures used (the predicted proportions from the logistic regression model with the events/trials dependent variable were allowed to take values anywhere between 0 and 1, but the outcome proportions could only take five values between 0 and 1). Or this might have been an artefact due to a large proportion of sampled houses with a low level of *Salmonella* in the litter detected.

Forcing the season of sampling or farm latitude into the fixed effects component of the final model (as the fourth fixed effects risk factor) was considered, but neither of these: i) allowed to overcome the trend in the residuals; ii) improved the model performance in terms of the three performance statistics used; or iii) demonstrated significant associations with the response (*p*≤0.050) given the other variables in the final model. Similar forcing of the farm longitude did not allow model convergence.

In the final model for the litter samples outcome (Table 2), none of the random effects factors appeared to significantly contribute to the variability observed in the response (Wald type test *p-*value>0.300 for each of the four factors).

### Development of final model of soil properties at broiler farm location associated with probability of detecting *Salmonella* in the drag swabs of litter

Of the numerical risk factors retained for the drag swabs outcome from the screening step of analysis, the percentage of soils of hydrologic group B in the map unit was pairwise collinear with both i) the ratio of the soils with comparably lower run-off potential to those with higher run-off potential, and ii) the proportion of the map unit components with perched water table. From these three variables, the percentage of soils of hydrologic group B (silt loam or loam) as a single fixed effects factor in the basic model had the smallest *p-*value and was preferred to keep for further modelling. Of the ordered risk factors retained from the screening for the drag swabs outcome, collinearity was observed between the suitability of the soils to produce habitat requirements for wetland wildlife, habitat for wetland plants, habitat element shallow water, and between these three variables and the soil's natural drainage class. Detailed soil properties were of primary interest in this analysis, therefore the natural drainage class variable, but not the other three, was kept for further modelling. As with the subset of risk factors used for building the final model for the litter samples outcome: i) the total water capacity of the soil profile and soil pH were collinear but both were preserved for further analysis; and ii) the soil capabilities to produce habitats for coniferous hardwood trees and woodland wildlife were completely collinear, and only the former was used further.

The risk factors remaining to build the final model for the drag swabs outcome were introduced into the basic model as fixed effects factors all at once. Convergence was not reached. Two variables with marginal significance at the screening level (*p*-value close to 0.150), namely, the soils' suitability to produce habitat for coniferous hardwood trees and the latest month when natural annual flooding could end in a normal year, as well as the next least significant variable: the overall clay content (*p* = 0.141), had to be removed before convergence was reached. Further variable selection (i.e. removal of the fixed effects factor with the highest *p-*value after each model fit until a model with all the fixed effects variables significant at *p*<0.050 was obtained) resulted in the full model that included three fixed effects risk factors (Table 2). The three factors were: i) the natural drainage class (for an improvement of one order OR = 0.13), ii) the percentage by weight of rock fragments greater than 7.6–25.4 cm in diameter (for each 0.25% increase OR = 1.06), and iii) the soil loss tolerance T-factor (for each 1 tonne increase OR = 0.05) for the soil map unit (Table 2). The Generalized Chi-Square/*df* for the full model was 0.77, the Spearman correlation coefficient between the observed and predicted responses was 0.80 (*p*<0.001), and the simple squared deviations statistic was 1.689.

Refinement of the full model in terms of reducing the number of fixed effects risk factors pursuing parsimony was considered. Removal of the soil loss tolerance variable resulted in a reduced model with a higher correlation between the observed and predicted responses (*ρ* = 0.81) compared to the full model, and a smaller squared deviations statistic of 1.67. The two risk factors remaining in this reduced model retained significance of associations with the response (*p*<0.050). The Generalized Chi-Square/*df* for this model was 0.84. This reduced model and the full model were further investigated with the diagnostic plots described in the preceding section. Compared to the full model, predictions from the reduced model tended to more greatly overestimate the response when the observed outcomes were ≤0.500. The reduced model also tended to underestimate the responses when the observed outcomes were >0.500. Non-parametric kernel density estimates of the Pearson-type residuals from the two models were plotted. The centre of the residuals' distribution appeared to be further to the left from zero for the reduced model than for the full model. The full model (Table 2) was adopted as the final model for the drag swabs outcome. When forced into the final model as the fourth fixed effects risk factor, neither the season of sampling, nor farm latitude or longitude was significantly (*p≤*0.050) associated with the response.

None of the four random effects factors in the final model (Table 2) appeared to make a significant contribution (Wald type test *p≤*0.050) to the variability in the drag swabs outcome. However, the contribution of the other differences among the farms was approaching such significance (*p = *0.104 for the farm effects; *p>*0.500 for each of the other three factors).
